# Hyperoside-Rich Blueberry Leaf Tea Improves Sleep Continuity in Adults with Poor Sleep: A Two-Week Randomized Double-Blind Controlled Trial

**DOI:** 10.3390/nu18030453

**Published:** 2026-01-30

**Authors:** Kentaro Shoji, Tomonori Yuasa, Yui Kitajima, Yoshiko Hirakawa

**Affiliations:** 1 Department of Food Science, Faculty of Home Economics, Otsuma Women’s University, 12 Sanbancho, Chiyoda-ku, Tokyo 102-8357, Japan; yuichon512@gmail.com; 2 Miyazaki Prefectural Food Research and Development Center, 16500-2 Higashi-Kaminaka, Sadowara-cho, Miyazaki 880-0303, Japan; yuasa-tomonori@pref.miyazaki.lg.jp (T.Y.); hirakawa.yoshiko.y4@miyazaki-u.ac.jp (Y.H.)

**Keywords:** blueberry leaf, hyperoside, polyphenol, sleep efficiency, actigraphy

## Abstract

Background/Objectives: Sleep is fundamental to physical and mental health, yet many individuals experience impaired sleep quality. Although pharmacological interventions are available, they are associated with risks of dependency and adverse effects, underscoring the urgent need for safer, food-based alternatives. Blueberry leaves, rich in hyperoside, are suggested to influence sleep through serotonergic and melatonergic pathways; however, while their potential to help maintain sleep quality has been noted, the sleep-enhancing effects of fermented blueberry leaf tea have not yet been demonstrated. This present randomized, double-blind, placebo-controlled trial evaluated the sleep-enhancing effects of fermented blueberry leaf tea on sleep quality. Methods: Fifty adults (aged 20–69 years) reporting poor sleep were randomly assigned to consume either fermented blueberry leaf tea (n = 25) or placebo tea (n = 25) three times daily for two weeks. Objective sleep parameters—sleep efficiency, wake after sleep onset (WASO), sleep latency, and total sleep time—were assessed using actigraphy, while subjective sleep quality was evaluated using the Oguri–Shirakawa–Azumi Sleep Inventory MA (OSA-MA) version questionnaire. Results: In the per-protocol analysis (active: n = 22; placebo: n = 20), the active group exhibited significant improvements in sleep efficiency and WASO compared with the placebo (*p* < 0.05). No significant differences were observed for sleep latency, total sleep time, or subjective assessments. Importantly, baseline sleep efficiency and WASO were negatively correlated with their respective improvements, suggesting that individuals with poorer initial sleep benefited most. Conclusions: These findings demonstrate that fermented blueberry leaf tea may enhance sleep continuity within two weeks, particularly among individuals with fragmented sleep, and support the potential role of functional foods in sleep health strategies. Trial registration: University Hospital Medical Information Network (UMIN), UMIN000055879; registered on 21 October 2024.

## 1. Introduction

Sleep is essential for maintaining overall health and well-being; however, sleep problems remain a major public health concern. Insufficient sleep affects an individual’s physical and mental health, quality of life, and daily activities. Studies have indicated that sleep disturbances, including insomnia and sleep apnea, are prevalent across various demographics and are influenced by factors such as age, sex, socioeconomic status, and cultural differences [1,2,3]. Sleep disorders contribute to serious health risks, including cardiovascular diseases, diabetes, obesity, and cognitive decline [4]. Additionally, poor sleep quality is closely linked to mental health issues such as depression and anxiety, exacerbating health disparities [5,6].

Given the limitations and potential adverse effects of pharmacological sleep treatments, functional foods have gained attention as safer, non-pharmacological approaches to improving sleep quality. Certain dietary components and bioactive compounds have been reported to modulate sleep through nutritional and biochemical mechanisms.

Zinc-rich foods, such as oysters and zinc-enriched yeast, have been shown to enhance sleep efficiency and reduce sleep onset latency, and astaxanthin further improves zinc absorption [7]. Fermented hispidin-enriched *Sanghuangporus sanghuang* mycelia (GKSS) have demonstrated sleep-promoting effects in animal studies by enhancing rapid eye movement (REM) and non-REM sleep through anti-inflammatory mechanisms [8].

Fruit- and plant-based extracts also play roles in sleep enhancement. Tart cherry juice, rich in melatonin, has been linked to improved sleep duration and quality [9,10,11]. Kiwifruit, containing high levels of antioxidants and serotonin, has shown similar benefits [9,11,12]. Additionally, honey has been suggested to have sleep-promoting effects; however, further research is required [13]. *Hibiscus syriacus* L. flower extract is currently being investigated for its potential sleep-enhancing properties [14].

Given this background, our previous interventional trial served as a pilot study to explore the sleep-improving effects of hyperoside, a flavonoid abundantly found in blueberry leaves [15]. Insights gained from this pilot study were used to refine the design of the present randomized, double-blind controlled trial, which aimed to investigate the effects of dietary fermented blueberry leaf tea on sleep quality under stable study conditions.

## 2. Material and Methods

### 2.1. Participants

This study enrolled healthy men and women between 20 and 69 years of age according to the predefined inclusion and exclusion criteria outlined in Table 1. Participants’ sleep quality was assessed using item C1 from the Japanese version of the Pittsburgh Sleep Quality Index (PSQI-J) [16], which asks: “During the past month, how would you rate your sleep quality overall?” After initial screening, exclusion criteria were applied to finalize participant eligibility. All individuals received a detailed explanation of the study objectives and procedures, and written informed consent was obtained under the supervision of the study director.

Eligible participants were stratified by sex, age, and PSQI-J score, and then randomly assigned to either the active or placebo group through stratified randomization.

### 2.2. Tea Preparation and Composition

The experimental tea, referred to as the “active tea”, was commercially produced from blueberry leaves that underwent oxidative fermentation to improve palatability while preserving the naturally high hyperoside content of the leaves [17]. The placebo tea was formulated from barley, with added citric acid and flavoring agents to closely match the macronutrient composition and caloric value of the active tea. Both teas were provided by Sunao Pharma, Inc. (Miyazaki, Japan).

The active tea was prepared using leaves from the rabbiteye blueberry cultivar ‘Kunisato 35 gou’. After harvesting, the leaves were immediately frozen at −20 °C. Subsequently, the frozen leaves were stored at 5 °C for 24 h to promote thawing and oxidative fermentation [18]. Following fermentation, the leaves were brought to room temperature (25 °C) and dried at 70 °C using a hot-air dryer until the moisture content was reduced to below 5%. These processed leaves were used to prepare the active tea.

To ensure double-blind conditions, both the active and placebo teas were placed into identical white tea bags, rendering them indistinguishable in taste, appearance, and the color of the brewed infusion.

The nutritional composition of each tea was analyzed by Kotobiken Medical Laboratories, Inc. (Tokyo, Japan) for energy, protein, fat, and carbohydrate content. Hyperoside content was quantified by Japan Food Research Laboratories (Tokyo, Japan) using high-performance liquid chromatography (HPLC) with a YMC-Pack ODS-A column (φ6.0 mm × 150 mm, particle size 5 µm, YMC Co., Ltd. (Kyoto, Japan) maintained at 40 °C. For sample preparation, three tea bags (total 6 g) were infused with 600 mL of boiling water and allowed to stand at room temperature for 3 min, after which the tea bags were removed. Approximately 1 g of the resulting infusion was accurately weighed, mixed with 15 mL of methanol, and subjected to ultrasonic extraction for 5 min. The extract was then brought to a final volume of 20 mL with methanol and used as the test solution for HPLC analysis. The mobile phase consisted of a mixture of water, acetonitrile, and 2-propanol (200:38:2) containing 0.4% citric acid, with a flow rate of 1.0 mL/min and detection at 360 nm.

### 2.3. Study Design and Ethics

The study was conducted in compliance with the ethical standards set forth in the Declaration of Helsinki and was approved by the Ethics Committee of Otsuma Women’s University (approval number: 06-009, issued on 1 August 2024). The trial protocol was registered in the University Hospital Medical Information Network Center (UMIN; registration number UMIN000055879; date of registration: 21 October 2024).

This randomized, double-blind, placebo-controlled, parallel-group trial consisted of a one-week pre-observation period followed by a two-week intervention phase during which participants consumed the assigned tea. The purpose of the pre-observation period was to assess baseline sleep status prior to the intervention, which is essential for accurately evaluating the effects of blueberry leaf tea on sleep outcomes. During this period, the participants’ sleep was monitored using the same actigraphy measurements and questionnaires applied during the intervention phase, following a predefined protocol. The average values obtained during the pre-observation period were used as baseline data for subsequent analyses.

A total of 50 healthy adults meeting the inclusion criteria were selected and randomized at baseline (week 0) into either the active or placebo group, using stratified randomization based on sex, age, and PSQI-J scores.

To minimize seasonal and environmental confounding, the study start was postponed from October to November due to extreme temperatures. Participants were instructed to maintain stable lifestyle habits throughout the study. Although ambient temperature, humidity, and day length were not continuously monitored, the intervention was conducted within a narrow seasonal window to reduce environmental variability.

The primary outcome was sleep efficiency measured by actigraphy. Secondary outcomes included wake after sleep onset (WASO), number of awakenings, and sleep latency assessed via actigraphy, as well as subjective sleep quality evaluated using the Oguri–Shirakawa–Azumi Sleep Inventory MA version (OSA-MA) questionnaire [19]. The OSA-MA was selected as a secondary outcome measure because its multidimensional structure allows for detailed evaluation of multiple aspects of subjective sleep quality during the intervention period.

### 2.4. Tea Consumption Protocol

Participants were instructed to prepare and consume the assigned tea three times daily using a standardized method.

To prepare each serving, one tea bag (2 g) was placed into an empty mug, and 200 mL of freshly boiled water was poured over the tea bag. The tea was allowed to steep for three minutes, after which the tea bag was removed without stirring or squeezing. Participants were advised to allow the tea to cool to a drinkable temperature before consuming it (Table 2).

### 2.5. Actigraphy Measurement

During the study period, participants wore an ActiGraph wGT3X-BT device (ActiGraph, Pensacola, FL, USA) on their non-dominant wrist, except during bathing or other activities involving water. The device recorded tri-axial acceleration data at a sampling rate of 30 Hz, with the data aggregated into 1-min epochs representing activity counts per minute.

Sleep and wake states were determined by analyzing the ActiGraph data using ActiLife software (version 6.13.3) and the Sadeh algorithm [20]. Sleep scoring was based on movement patterns recorded in 1-min epochs, with the participants’ reported bedtimes and wake-up times manually entered into the software to enhance accuracy.

The following six sleep parameters were derived from actigraphy during the main sleep period:

1. Sleep efficiency: Percentage of time spent asleep, calculated as total sleep time divided by time in bed.
2. Wake after sleep onset (WASO): The cumulative time spent awake after initially falling asleep.
3. Average awakening length: The average duration of wake periods during sleep.
4. Number of awakenings: The total number of wake episodes during sleep.
5. Sleep latency: The duration from bedtime to the onset of sleep.
6. Total sleep time: The cumulative duration of epochs scored as sleep throughout the sleep period.

### 2.6. Subjective Sleep Assessment

Upon awakening each morning, participants completed the Oguri–Shirakawa–Azumi Sleep Inventory MA version (OSA-MA) to assess their subjective sleep quality.

The OSA-MA consists of 16 standardized questions categorized into five factors:

1. Sleepiness upon waking;
2. Sleep onset and maintenance;
3. Dream experiences;
4. Recovery from fatigue;
5. Sleep duration.

Responses were scored on a four-point Likert scale (ranging from 1 to 4).

Subscale scores were calculated using the standardized OSA-MA scoring program, and results were expressed as corrected Zc scores, with higher Zc scores indicating better subjective sleep quality.

### 2.7. Lifestyle and Compliance Monitoring

Throughout the intervention period, participants maintained a daily record to document their tea intake, eating habits, exercise activities, and overall physical condition, using a simple checklist format.

They were instructed to maintain their usual dietary and lifestyle routines, refrain from excessive eating or drinking, and avoid major changes to their exercise or smoking habits in order to minimize potential confounding factors.

### 2.8. Statistical Analysis

The primary analysis was performed on the Per Protocol Set (PPS). In sleep research, sleep parameters are highly sensitive physiological phenomena that can be significantly influenced by external confounding factors other than the intervention components. For instance, acute stressors, alcohol consumption, or environmental noise can cause substantial night-to-night variability in sleep data [21,22]. Furthermore, when using actigraphy for objective assessment, irregular lifestyles—such as business trips or non-compliance with device wearing—can render the physiological effects of the intervention difficult to interpret [23]. Therefore, to minimize these external “noises” and to rigorously evaluate the intrinsic efficacy (the biological effect) of the fermented blueberry leaf tea, we pre-specified the PPS as the primary population for all efficacy analyses. This approach aligns with the principles of the ICH-E9 guideline (1998) [24] and its E9(R1) addendum (2019) [25], which emphasize the importance of defining an “estimand” that accurately reflects the treatment effect under strict adherence to the protocol conditions.

Sample size was determined based on the effect size observed in our previous trial involving a similar population. To ensure the reproducibility of the primary outcome under identical experimental conditions, a total of 50 participants were enrolled. This sample size was considered appropriate for evaluating the primary outcome under the same experimental conditions, in line with the design of our previous pilot study [15].

Sleep parameters obtained from actigraphy and OSA-MA scores were averaged over each one-week period. Data are expressed as means with standard errors (mean ± SE). Values of actigraphic measurement data and OSA-MA scores were expressed as measured values and changes (Δ) from the initial values. To assess the primary and secondary outcomes, significant differences were assessed using a linear mixed-effects model with fixed effects for treatment and time interaction. Post hoc comparisons between groups at each time point were conducted using the Bonferroni correction to account for multiple comparisons. As an additional analysis, the correlation between the baseline values and the changes from baseline at 2 weeks (Δ) for both actigraphic measurement data and OSA-MA scores was assessed using Pearson’s correlation coefficient in the active group. All the statistical analyses were performed using SPSS Statistics version 27.0 (IBM Inc., Armonk, NY, USA). The significance level was set at 5% (two-sided), with *p* < 0.05 considered statistically significant.

## 3. Results

### 3.1. Participant Flow and Baseline Characteristics

**Figure 1:**
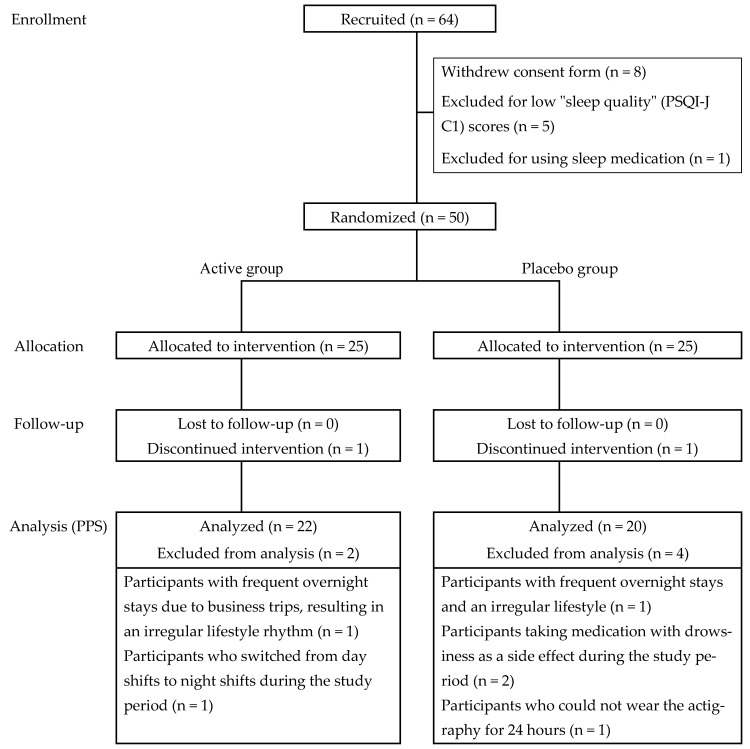
Participant flowchart for the study. The number of participants at each stage of the study is shown.

A total of 64 participants were recruited, of whom eight withdrew consent, five were excluded because of low PSQI-J C1 scores, and one was excluded for using sleep medication. Fifty eligible participants were randomly assigned to either the active (n = 25) or placebo group (n = 25) (Figure 1). During the intervention, one participant in the active group withdrew due to a dislike of the taste of the test tea, and one in the placebo group withdrew due to work-related constraints preventing continued actigraphy use. This left 24 participants in each group for the follow-up. For the per-protocol set analysis, data from 22 participants in the active group and 20 in the placebo group were analyzed after additional exclusions. In the active group, one participant was excluded due to frequent business trips causing an irregular lifestyle and another due to a shift change from day to night work. In the placebo group, one participant was excluded because of frequent overnight stays and an irregular lifestyle, two for taking medication with drowsiness as a side effect, and one for not being able to wear the actigraphy for 24 h. Baseline characteristics, including age, sex distribution, height, weight, body mass index, and PSQI-J scores, showed no significant differences between the groups (Table 3).

### 3.2. Objective Sleep Quality Measured Using Actigraphy

Sleep efficiency (Table 4) significantly increased, and WASO (Table 4) significantly decreased in the active group, both demonstrating a treatment-by-time interaction compared with the placebo group. Sleep efficiency increased from 80.7 ± 1.3% at baseline to 83.7 ± 1.1% at week 2 in the active group, whereas no significant change was observed in the placebo group (81.2 ± 2.0% at baseline to 81.8 ± 2.0% at week 2). The change in sleep efficiency (Δ sleep efficiency) was significantly greater in the active group compared with the placebo group (3.0 ± 0.8% vs. 0.6 ± 0.6%, *p* < 0.05) (Table 4).

Similarly, WASO significantly decreased in the active group from 74.7 ± 6.3 min at baseline to 59.7 ± 5.2 min at week 2, whereas no significant reduction was observed in the placebo group (76.7 ± 9.5 min at baseline to 73.7 ± 9.0 min at week 2). The change in WASO (Δ WASO) was significantly more pronounced in the active group (−15.1 ± 3.6 min vs. −3.1 ± 2.9 min, *p* < 0.05).

**Figure 2:**
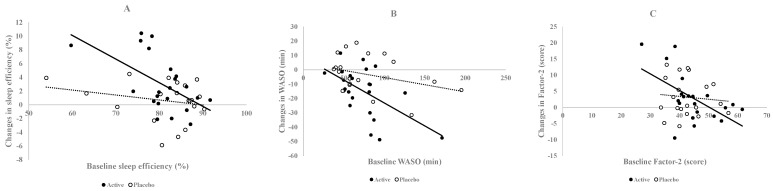
Correlations between changes in sleep parameters and baseline sleep parameters in the active group (closed circle and solid line) and placebo group (open circle and broken line). (**A**) Sleep efficiency (active: r = −0.550, *p* = 0.008, placebo: r = −0.234, *p* = 0.321), (**B**) WASO (active: r = −0.562, *p* = 0.007, placebo: r = −0.328, *p* = 0.159), and (**C**) Factor-2 (Sleep onset and maintenance) (active: r = −0.557, *p* = 0.009, placebo: r = −0.096, *p* = 0.688).

Furthermore, a significant negative correlation was observed between baseline sleep efficiency and its change after 2 weeks (r = −0.550, *p* = 0.008) and between baseline WASO and its change (r = −0.562, *p* = 0.007) in the active group, indicating that participants with lower baseline sleep efficiency and higher baseline WASO experienced greater improvements (Figure 2A,B).

No significant differences were observed between the groups in terms of the number of awakenings, average awakening length, sleep latency, or total sleep time throughout the study period.

### 3.3. Subjective Sleep Quality Assessed Using the OSA-MA

OSA-MA scores showed no significant differences between the active and placebo groups across all five factors (Table 5). However, a significant negative correlation was observed between baseline factor-2 (Sleep onset and maintenance) and its change after 2 weeks (r = −0.550, *p* = 0.008) in the active group, indicating that participants with lower baseline factor-2 experienced greater improvements (Figure 2C).

### 3.4. Compliance and Safety

Throughout the 2-week intervention period, participants in both the active and placebo groups demonstrated high compliance, with recorded intake rates of 98.8% and 98.3%, respectively.

No adverse events or discomfort were reported in association with the consumption of either tea, indicating that both were well tolerated.

## 4. Discussion

The present study demonstrated that the consumption of blueberry leaf tea significantly improved sleep efficiency and WASO, as assessed using actigraphy, whereas no significant changes were observed in sleep latency or total sleep time. Numerous studies have emphasized the importance of improving sleep efficiency and WASO over merely increasing the total sleep time to optimize sleep quality and overall health outcomes.

Cognitive behavioral therapy for insomnia has been shown to significantly enhance sleep efficiency and reduce WASO, reinforcing the idea that these metrics are crucial for effective insomnia treatment [26,27]. Similarly, studies on adolescent sleep interventions have reported that cognitive-behavioral sleep strategies yield greater improvements in sleep efficiency and WASO than in total sleep time, suggesting that these parameters play a more significant role in adolescent sleep health [28]. Additionally, research on app-supported sleep coaching found that individuals with initially low sleep efficiency exhibited greater enhancements in sleep efficiency and reductions in WASO compared with changes in total sleep time, highlighting the importance of prioritizing sleep efficiency improvements in digital sleep interventions [29].

Furthermore, studies on herbal sleep supplements, such as ashwagandha, indicated that while increases in total sleep time were observed, the primary benefits of supplementation were better sleep efficiency and WASO, suggesting that improvements in these parameters are more directly associated with enhanced sleep quality [30]. Moreover, research examining socioeconomic disparities in sleep suggests that neighborhood disadvantages disproportionately affect WASO more than total sleep time, further emphasizing the role of sleep continuity in overall sleep health [31].

Taken together, these findings suggest that sleep efficiency and WASO should be the primary focus of sleep interventions. The present study’s results support this perspective, demonstrating that blueberry leaf tea consumption effectively improves sleep maintenance rather than merely extending sleep duration. Future research should continue to investigate the mechanisms underlying sleep efficiency and WASO improvements and explore how these findings can be applied to broader populations and clinical settings.

In this study, the subjective OSA-MA endpoint did not show significant changes despite clear improvements in objective sleep continuity parameters assessed by actigraphy. This dissociation between subjective and objective sleep outcomes is a well-recognized phenomenon in sleep research, often referred to as subjective–objective sleep discrepancy or sleep misperception.

Recent large-scale real-world studies using in-home sleep electroencephalography have demonstrated that subjective sleep assessments are insufficient for detecting nocturnal awakenings and sleep fragmentation. Masaki et al. reported that although subjective sleep quality partially reflects macro sleep architecture such as sleep efficiency, it does not reliably capture frequent short awakenings or objective sleep instability [32]. Importantly, improvements in objective parameters such as WASO were often not consciously perceived by the participants, highlighting the limitations of questionnaire-based tools for evaluating sleep continuity.

In addition, the OSA-MA assesses not only nocturnal sleep but also broader dimensions such as lifestyle factors, stress, and daytime functioning, which may require a longer intervention period to improve [19]. Taken together, the lack of subjective improvement in OSA-MA scores should not be interpreted as contradictory, but rather as physiologically plausible and consistent with accumulating evidence emphasizing the importance of objective sleep assessments.

Blueberry leaves are rich in polyphenols, including catechins, chlorogenic acid, and hyperoside. Among these compounds, chlorogenic acid and hyperoside have been reported to directly improve sleep quality. According to the study by Ochiai et al. [33], the sleep-enhancing effect of chlorogenic acid was observed at a daily dose of 300 mg. However, in the present blueberry leaf sample, the contents of chlorogenic acid derivatives were 3.8 mg of 3-caffeoylquinic acid and 24.9 mg of 5-caffeoylquinic acid, indicating that it is unlikely that the observed improvement in sleep was attributable to these compounds. In contrast, hyperoside has been shown to improve sleep even at a low dose of 1.0 mg per day [34], as demonstrated in clinical trials using subjective sleep quality measures such as the OSA-MA. Given that the sample used in the present study contained 5.4 mg of hyperoside, it is highly likely that hyperoside contributed to the observed improvement in sleep quality.

The present study demonstrated that the consumption of blueberry leaf tea significantly improved sleep efficiency and WASO within a short intervention period of two weeks, suggesting a potential short-term benefit on sleep continuity.

Although the underlying mechanisms remain unclear, one possible explanation involves the modulation of serotonin–melatonin-related pathways. Blueberry leaf tea contains hyperoside, which has been reported to be metabolized into quercetin and may influence monoamine oxidase activity, potentially affecting serotonin metabolism [35]. Previous pharmacokinetic studies have reported that quercetin-derived conjugates, such as quercetin-3-O-β-glucuronide, are detectable in brain tissue in animal models, suggesting a potential for limited blood–brain barrier permeability [36]. However, as neither plasma nor brain concentrations of hyperoside or its metabolites were measured in the present study, any discussion of central mechanisms should be regarded as speculative. Nonetheless, since serotonin serves as a precursor to melatonin, alterations in serotonin availability could, in theory, influence the regulation of sleep–wake cycles and nocturnal sleep maintenance.

Previous experimental studies have shown that serotonin plays an important role in sleep and circadian regulation in animal models [37,38,39,40]. Additionally, in this clinical trial, blueberry leaf tea was consumed thrice a day, which may have contributed to the sustained availability of serotonin throughout the day, further supporting the observed improvements in sleep efficiency and WASO through the gradual modulation of melatonin secretion. These interpretations remain tentative, as neither serotonin nor melatonin levels were directly measured in the present study. Future studies incorporating direct biochemical measurements will be necessary to clarify the precise mechanisms underlying the sleep-related effects of blueberry leaf tea.

Furthermore, sleep efficiency and WASO are parameters that respond more quickly to physiological changes than other sleep indices, such as sleep latency and total sleep time. A meta-analysis of brief behavioral treatments for insomnia found significant improvements in sleep efficiency and WASO at early follow-up, whereas sleep latency and total sleep time showed less consistent improvements [41]. Reducing WASO directly increases sleep efficiency by minimizing night wakefulness, making it a critical target for sleep interventions. Given that WASO significantly decreased in this study, it is plausible that blueberry leaf tea contributed to sleep consolidation, thereby improving sleep efficiency over a relatively short period.

Furthermore, the rapid improvement in sleep efficiency and WASO may have been influenced by the baseline sleep characteristics of the participants. Individuals with lower initial sleep efficiency or higher WASO may have responded more to the intervention, leading to more pronounced improvements over 2 weeks. This is supported by the negative correlation between baseline sleep efficiency and WASO and their respective changes after 2 weeks of active tea consumption in the present study (Figure 2).

Overall, these findings indicate that blueberry leaf tea may have a beneficial impact on sleep maintenance through serotonin–melatonin modulation, leading to measurable improvements in sleep efficiency and WASO even within a short intervention period. Future research should explore longer intervention durations and objective serotonin level measurements to further elucidate the underlying mechanisms and assess the long-term efficacy of blueberry leaf tea in improving sleep quality.

## 5. Study Limitations

One limitation of this study is that while significant improvements were observed in sleep efficiency and WASO, as assessed using actigraphy, no significant changes were detected in the OSA-MA scores. This discrepancy may be attributed to the relatively short 2-week intervention period, which may not have been sufficient for the participants to perceive subjective improvements in sleep quality. While objective sleep parameters, such as sleep efficiency and WASO, can respond more rapidly to physiological changes, subjective sleep assessments may require a longer adaptation period to reflect meaningful improvements. Future studies should consider extending the intervention duration to further evaluate the long-term effects of blueberry leaf tea on objective and subjective sleep quality.

In addition, this study did not include direct biochemical measurements of serotonin or melatonin. Further studies incorporating biochemical assessments will be necessary to clarify the underlying mechanisms of the sleep-related effects of blueberry leaf tea.

## 6. Conclusions

This study demonstrated that the consumption of blueberry leaf tea significantly improved sleep efficiency and WASO, as assessed using actigraphy, suggesting its potential efficacy in enhancing sleep continuity.

Improving sleep quality is essential for overall well-being, as better sleep contributes to enhanced cognitive function, emotional stability and daytime performance. The findings of this study suggest that blueberry leaf tea consumption may be beneficial for individuals experiencing sleep fragmentation or poor sleep maintenance, ultimately supporting improved daily functioning and quality of life. Future studies should consider longer intervention periods and comprehensive sleep assessments to further evaluate the long-term benefits on sleep and overall health.

## Figures and Tables

**Table 1:** Criteria for inclusion and exclusion.

** Inclusion criteria **
Healthy male and female adults aged 20 to 69 years who reported experiencing poor sleep quality.
** Exclusion criteria **
Participants were excluded if they met any of the following conditions:
1. Current or past diagnosis of respiratory, gastrointestinal, hepatobiliary, hematologic, renal, endocrine, or cardiovascular disorders.
2. History of major trauma or surgery within 12 weeks prior to study enrollment.
3. History or suspicion of allergic diseases requiring medical treatment for food or drug allergies.
4. Regular consumption of blueberry leaf tea as identified during screening by the study team.
5. Active engagement in activities aimed at improving sleep or managing fatigue and stress at the time of screening.
6. Frequent nighttime awakenings, such as due to nocturia.
7. Diagnosis of sleep apnea syndrome or chronic fatigue syndrome.
8. Known allergy to components of the test tea.
9. Participation in other clinical trials within four weeks prior to study commencement.
10. Any other condition deemed inappropriate for participation by the principal investigator.

**Table 2:** Nutrient composition of the active and placebo teas (200 mL).

	Active	Placebo
Energy (kcal)	2.0	0.0
Protein (g)	0.0	0.0
Fat (g)	0.0	0.0
Carbohydrate (g)	0.6	0.2
Hyperoside (mg)	1.8	0.0

**Table 3:** Participant Characteristics.

	Active (n = 22)			Placebo (n = 20)			*p*-Value
Sex (Female/Male)	12/10			10/10			
Age (years)	40.6	±	2.8	41.5	±	3.1	0.848
Height (cm)	164.6	±	2.4	164.5	±	2.0	0.978
Weight (kg)	60.3	±	3.0	61.0	±	2.7	0.862
Body mass index (kg/m^2^)	22.0	±	0.6	22.4	±	0.6	0.703
PSQI-J score	5.7	±	0.5	5.9	±	0.4	0.792

Values are means ± SE.

**Table 4:** Changes in Sleep Parameters by Actigraphy.

		0 Week			1 Week			2 Weeks			*p*-Value
Sleep efficiency	Active	80.7	±	1.3	81.5	±	1.3	83.7	±	1.1	0.036
(%)	Placebo	81.2	±	2.0	81.8	±	2.1	81.8	±	2.0	
	Δ Active	0.0	±	0.0	0.8	±	1.1	3.0	±	0.8 *	0.042
	Δ Placebo	0.0	±	0.0	0.5	±	0.6	0.6	±	0.6	
Wake time after sleep onset	Active	74.7	±	6.3	70.3	±	6.3	59.7	±	5.2	0.015
(min)	Placebo	76.7	±	9.5	74.0	±	9.7	73.7	±	9.0	
	Δ Active	0.0	±	0.0	−4.4	±	4.6	−15.1	±	3.6 *	0.021
	Δ Placebo	0.0	±	0.0	−2.7	±	2.6	−3.1	±	2.9	
Number of awakenings	Active	20.0	±	1.2	18.3	±	1.5	17.4	±	1.4	0.070
(times)	Placebo	21.6	±	1.3	20.8	±	1.5	21.0	±	1.3	
	Δ Active	0.0	±	0.0	−1.7	±	0.8	−2.6	±	0.7	0.188
	Δ Placebo	0.0	±	0.0	−0.8	±	0.8	−0.6	±	0.7	
Average awakening length	Active	3.8	±	0.2	4.2	±	0.3	3.8	±	0.4	0.108
(min)	Placebo	4.0	±	0.5	3.4	±	0.2	3.4	±	0.2	
	Δ Active	0.0	±	0.0	0.4	±	0.3	0.0	±	0.4	0.205
	Δ Placebo	0.0	±	0.0	−0.6	±	0.4	−0.6	±	0.4	
Sleep latency	Active	4.9	±	0.7	5.0	±	0.8	4.2	±	1.0	0.456
(min)	Placebo	6.6	±	0.9	5.2	±	0.7	6.5	±	1.2	
	Δ Active	0.0	±	0.0	0.0	±	1.0	−0.7	±	0.9	0.253
	Δ Placebo	0.0	±	0.0	−1.4	±	0.8	−0.1	±	1.1	
Total sleep time	Active	325.3	±	8.0	327.2	±	10.5	323.9	±	8.6	0.725
(min)	Placebo	354.3	±	10.7	350.2	±	10.6	354.2	±	10.5	
	Δ Active	0.0	±	0.0	1.9	±	9.3	−1.4	±	6.0	0.426
	Δ Placebo	0.0	±	0.0	−4.1	±	6.2	−0.1	±	5.4	

Data are means  ±  SE (n = 42). Δ indicates the change from baseline. Treatment-by-time interactions were assessed separately for each actigraphic sleep parameter using a linear mixed model, with *p* values shown in the table. Significantly different from the placebo tea in Bonferroni’s multiple comparison test: * *p* < 0.05.

**Table 5:** Changes in Sleep Parameters by OSA-MA Questionnaire.

		0 Week			1 Week			2 Weeks			*p*-Value
Factor 1	Active	43.8	±	2.0	43.7	±	2.0	44.6	±	2.0	0.867
(Sleepiness upon waking)	Placebo	44.8	±	1.4	45.2	±	1.6	46.4	±	1.3	
	Δ Active	0.0	±	0.0	−0.1	±	1.3	0.8	±	0.9	0.855
	Δ Placebo	0.0	±	0.0	0.4	±	0.7	1.6	±	1.2	
Factor 2	Active	44.9	±	1.7	46.2	±	1.5	47.7	±	1.6	0.977
(Sleep onset and maintenance)	Placebo	42.6	±	1.4	44.1	±	1.8	45.8	±	1.8	
	Δ Active	0.0	±	0.0	1.4	±	1.3	2.8	±	1.6	0.850
	Δ Placebo	0.0	±	0.0	1.4	±	1.1	3.2	±	1.3	
Factor 3	Active	49.2	±	1.3	51.1	±	1.5	50.6	±	1.5	0.548
(Dreaming)	Placebo	44.5	±	1.9	46.0	±	2.0	46.9	±	1.6	
	Δ Active	0.0	±	0.0	1.8	±	1.0	1.3	±	1.1	0.278
	Δ Placebo	0.0	±	0.0	1.5	±	0.9	2.4	±	1.2	
Factor 4	Active	46.0	±	1.9	46.6	±	1.9	45.8	±	1.9	0.378
(Restoration from fatigue)	Placebo	45.0	±	1.5	46.1	±	1.5	47.1	±	1.5	
	Δ Active	0.0	±	0.0	0.6	±	1.5	−0.2	±	0.9	0.352
	Δ Placebo	0.0	±	0.0	1.1	±	1.0	2.1	±	1.3	
Factor 5	Active	43.1	±	1.6	43.9	±	1.8	44.2	±	1.6	0.806
(Sleep duration)	Placebo	46.3	±	1.4	47.0	±	1.3	48.4	±	1.4	
	Δ Active	0.0	±	0.0	0.8	±	1.0	1.2	±	1.0	0.540
	Δ Placebo	0.0	±	0.0	0.6	±	0.9	2.0	±	1.1	

Data are means  ±  SE (n = 41). Δ indicates the change from baseline. OSA-MA data were available for 41 participants due to one missing questionnaire during the intervention period. Treatment-by-time interactions were assessed separately for each OSA-MA parameter using a linear mixed model, with *p* values shown in the table.

## Data Availability

The participants in this study did not provide written consent for public data sharing; therefore, due to the sensitive nature of the research, supporting data were not available.
